# Ophthalmic Use of Targeted Biologics in the Management of Intraocular Diseases: Current and Emerging Therapies

**DOI:** 10.3390/antib13040086

**Published:** 2024-10-11

**Authors:** Yuan Zong, Miki Miyagaki, Mingming Yang, Jing Zhang, Yaru Zou, Kyoko Ohno-Matsui, Koju Kamoi

**Affiliations:** Department of Ophthalmology & Visual Science, Graduate School of Medical and Dental Sciences, Tokyo Medical and Dental University, Tokyo 113-8510, Japan; zongyuan666@gmail.com (Y.Z.); miyagakimk@gmail.com (M.M.); yangmm-12@outlook.com (M.Y.); zhangj.c@foxmail.com (J.Z.); alicezouyaru519@gmail.com (Y.Z.); k.ohno.oph@tmd.ac.jp (K.O.-M.)

**Keywords:** monoclonal antibodies (mAbs), anti-VEGF therapy, intraocular diseases, non-infectious uveitis, age-related macular degeneration (AMD), retinal vein occlusion (RVO), diabetic retinopathy, complement system

## Abstract

Background: Monoclonal antibodies (mAbs) have demonstrated substantial potential in the treatment of intraocular diseases. This review aimed to comprehensively evaluate the applications, efficacy, and safety of mAbs in the management of intraocular conditions. Methods: A comprehensive literature search was conducted in major medical databases through July 2024. Relevant studies on monoclonal antibodies for intraocular diseases were included. Two independent researchers screened the literature, extracted data, and assessed study quality. Cost-effectiveness analyses were also reviewed. Results: Anti-vascular endothelial growth factor (VEGF) antibodies, such as bevacizumab, ranibizumab, and aflibercept, showed significant therapeutic effects in neovascular age-related macular degeneration (NVAMD), diabetic macular edema (DME), and retinal vein occlusion (RVO). Tumor necrosis factor-alpha (TNF-α) inhibitors demonstrated promising results in treating noninfectious uveitis. Complement system-targeted therapies like pegcetacoplan offered new options for geographic atrophy. Anti-VEGF antibodies showed potential in managing retinopathy of prematurity (ROP). However, challenges persist, including high costs, potential drug resistance, and limited long-term safety data in certain scenarios. Conclusions: Monoclonal antibodies are vital for treating intraocular diseases, but continuous innovation and rigorous clinical evaluation are essential. Future research should focus on developing novel delivery systems, exploring combination therapies, conducting long-term follow-up studies, and investigating personalized treatment strategies to provide safer, more effective, and cost-effective therapeutic solutions.

## 1. Introduction

Antibodies are a critical subset of the glycoprotein class in the immunoglobulin (Ig) superfamily and act as vital components within the immune system by targeting foreign antigens for identification and neutralization to induce myriad immune responses. Monoclonal antibodies (mAbs) are homogeneous populations of immunoglobulins derived from a single B-cell clone that exhibit exquisite specificity for distinct epitopes on the target antigens [1]. The use of mAbs makes it possible to pinpoint particular antigens and achieve high binding affinity to target abnormal tissues while reducing off-target effects in normal tissue [1,2,3]. The initial success of mAbs in cancer treatment was primarily due to their unparalleled specificity, which has consequently led to other clinical applications including ophthalmology. mAbs are promising in the ophthalmic field, as recently presented investigations have shown their efficacy in treating different ocular conditions refractory to classic therapeutic options. The advent of mAbs has revolutionized the ophthalmic therapeutic space, providing a unique paradigm for patients suffering from sight-threatening conditions.

Intraocular diseases, such as uveitis and neovascular age-related macular degeneration (NVAMD), are among the most frequent causes of visual impairment and blindness worldwide, affecting millions of people [4]. Strategies for the treatment of these conditions typically include corticosteroids, immunosuppressive agents, laser therapy, and surgery, with variable health outcomes [4]. These disease states not only negatively affect the lives of patients but also represent a significant economic burden on healthcare systems. Standard restorative interventions for these conditions, consisting of corticosteroids, immunosuppressive agents, laser treatment, and surgical procedures, are commonly used but do not provide significant advantages in the long term, and they also have their own harmful impacts [3,5,6,7]. mAbs have introduced the concept of a mechanism of action mediated by the selective inhibition of pathways involved in these diseases, which presents more specific and potent treatment options.

The application of mAbs in ophthalmology has led to significant therapeutic improvements over the past decade, with one of the greatest successes being the development of anti-vascular endothelial growth factor (anti-VEGF) therapies. These mAbs were first approved for use in NVAMD and later for proliferative diabetic retinopathy, both of which are diseases associated with abnormal blood vessel growth and excessive vascular permeability, which result in impaired retinal perfusion. This breakthrough has revolutionized the treatment of various retinal disorders characterized by pathological angiogenesis and vascular leakage [8]. Ranibizumab is the first U.S. Food and Drug Administration (FDA)-approved mAb fragment against VEGF for use in ophthalmology [9]. In clinical trials, ranibizumab demonstrated significant improvements in visual acuity; patients achieved an average gain of 10.3 letters on the Safety and Efficacy of Ranibizumab in Diabetic Macular Edema (RESOLVE Study) chart at 12 months [10]. Following the success of ranibizumab, newer agents such as aflibercept and brolucizumab have been developed, offering additional benefits, such as extended dosing intervals and improved patient compliance [11,12]. These advancements emphasize the potential of mAbs to transform the management of ocular diseases, offering clinicians and patients more efficient and convenient treatment options.

In addition to the anti-VEGF therapies, mAbs targeting inflammatory pathways have shown promise in treating uveitis and other inflammatory ocular conditions. Adalimumab, an anti-tumor necrosis factor (TNF) mAb, has been approved for the treatment of noninfectious uveitis, providing an alternative for patients who are unresponsive to conventional immunosuppressive therapies [13,14,15]. Table 1 summarizes the mAbs approved by the FDA for the treatment of intraocular diseases along with their specific indications and mechanisms of action.

In this review, we discuss the current state of mAb therapies for intraocular diseases and examine their mechanisms of action, clinical efficacy, and future prospects. By providing a comprehensive overview, we aim to elucidate the role of mAbs in advancing ophthalmic care and highlight ongoing innovations that promise to further refine these therapeutic strategies.

## 2. Materials and Methods

This review aimed to comprehensively evaluate the application of mAbs in the management of intraocular diseases. An extensive literature search encompassing relevant studies from inception through July 2024 was performed. The electronic databases searched included Medline, PubMed, the Science Citation Index within the Web of Science, and Cochrane Library. The search terms employed comprised “monoclonal antibody”, “anti-VEGF”, “anti-TNF-α”, and “complement inhibitor”, utilized both individually and in conjunction with terms such as “uveitis”, “age-related macular degeneration”, “diabetic retinopathy”, and “retinopathy of prematurity” and descriptors such as “efficacy”, “safety”, “pharmacokinetics”, and “cost-effectiveness”.

Original research articles published in English, systematic reviews, meta-analyses, and pivotal clinical trials were included.

The inclusion criteria were as follows: (1) studies addressing the use of mAbs in the treatment of intraocular diseases; (2) reports detailing pharmacodynamics, clinical efficacy, or safety data; and (3) human studies or critical animal model investigations. The exclusion criteria were as follows: (1) non-English literature; (2) case reports; and (3) duplicate publications.

Two independent researchers conducted the literature screening and data extraction; any discrepancies were resolved through discussion or consultation with a third-party expert. Extracted data included the study design, sample size, interventions administered, follow-up duration, primary and secondary outcome measures, and adverse events. For the included clinical trials, we assessed the methodological quality based on randomization methods, implementation of blinding procedures, attrition rates, and other factors.

Furthermore, we searched for pertinent health economics studies to evaluate the cost-effectiveness of monoclonal antibody therapies. Through a comprehensive analysis of these findings, we aimed to provide a thorough and objective overview of the current status and future directions concerning the application of mAbs in managing intraocular diseases.

## 3. Monoclonal Antibody Therapy for Uveitis

### 3.1. Uveitis

Uveitis is an ocular inflammatory condition that primarily affects the uveal tract, which comprises the iris, ciliary body, and choroid [16]. This disease can present with a variety of symptoms including ocular pain, blurred vision, eye redness, and photophobia [7]. Uveitis may result in complications that threaten vision, such as cataracts, glaucoma, and retinal detachment, and is recognized as the fifth leading cause of vision loss in developed countries [17,18]. The etiology of uveitis is complex and involves a diverse array of factors that distinguish infectious uveitis from noninfectious uveitis (NIU) [6,16,19]. Depending on the location and nature of inflammation, uveitis can be classified into anterior, intermediate, posterior, and pan-uveitis [6,16,20].

Historically, patients with NIU have primarily relied on the use of corticosteroids, either topically or systemically, for treatment, particularly during acute episodes [16]. However, the prolonged use of corticosteroids may lead to side effects and other ocular complications [16]. In cases where patients with NIU have not successfully controlled intraocular inflammation after more than three months of systemic treatment with ≤5 mg/day prednisone, or when they are intolerant to corticosteroids and/or face a significant threat to their vision, immunomodulatory therapy is initiated [3,21]. For patients with uveitis refractory to conventional treatments, biological agents have demonstrated not only significant therapeutic efficacy but also a validated safety profile [16].

### 3.2. Anti-TNF-α Monoclonal Antibodies

Tumor necrosis factor-alpha (TNF-α) is a pro-inflammatory cytokine primarily produced by macrophages, T cells, and other immune cells. It plays a critical role in mediating the inflammatory and immune responses. In the context of uveitis, TNF-α contributes to disruption of the blood–ocular barrier, recruitment of inflammatory cells, and exacerbation of intraocular tissue damage [6,14,22,23]. Research has demonstrated that TNF-α levels are elevated in the aqueous humor and vitreous fluid of patients with uveitis, correlating with the severity of the disease [24,25]. Anti-TNF mAbs were designed to neutralize the activity of TNF-α, thereby reducing inflammation and preventing tissue damage. These biologics have shown significant efficacy in treating noninfectious posterior uveitis and pan-uveitis. However, their effectiveness in anterior uveitis is comparatively limited [26]. Two commonly used anti-TNF mAbs for the treatment of noninfectious uveitis are adalimumab (ADA) and IFX [7,13].

#### 3.2.1. Adalimumab

ADA is a fully human monoclonal antibody that binds to TNF-α and prevents its interaction with receptors on the surface of target cells (TNFR1 and TNFR2) [7,27,28]. In 2016, ADA was approved by the U.S. and Japan as the first anti-TNF-α agent for NIU treatment [29]. In the prospective randomized studies VISUAL I and VISUAL II, ADA demonstrated significant efficacy in the treatment of NIU, particularly in terms of swift management of inflammation, prevention of recurrences, and decreased reliance on corticosteroids [13,30]. In the subsequent VISUAL III study, 371 patients who met the treatment failure (TF) criteria or completed VISUAL I or II without TF were included, of whom 242 had active uveitis. At the end of the study (78 weeks), 60% (145/242) of patients with active uveitis achieved quiescence, while 40% (97/242) did not reach quiescence [31]. A meta-analysis conducted in 2018 incorporated and evaluated three randomized clinical trials (RCTs) and 20 non-randomized studies [32]. Among patients who were “almost naïve” to anti-TNF-α therapy, the rate of inflammation control after initiating ADA treatment was 87% (95% CI 80–92%). Furthermore, 41.3% (52/126) of eyes exhibited an improvement of three lines or more in visual acuity, and 88.8% (142/160) maintained visual acuity that was equal to or better than the baseline measurements. Additionally, a significant reduction in corticosteroid dependency was noted among 82.0% (91/111) of patients; notably, 48.8% (40/82) were able to completely discontinue corticosteroid use. The therapeutic efficacy of ADA remained consistently favorable across the three RCTs, with ADA demonstrating a reduction in the risk of treatment failure ranging from 43% to 75% [32].

#### 3.2.2. Infliximab

IFX is a chimeric monoclonal antibody composed of a human constant region and murine variable region. In addition to its ability to bind both soluble and membrane-bound forms of TNF-α, infliximab induces apoptosis in TNF-α-producing cells, further contributing to the reduction of inflammation [15,28]. Since its initial application in uveitis in 2001, IFX has emerged as one of the most frequently used biological therapies for NIU [29,33]. A retrospective study involving patients with refractory noninfectious uveitis reported a complete clinical remission rate of 82% (72/88) after treatment with IFX [34]. A meta-analysis of 88 studies involving a cohort of 369 patients with Behçet’s disease treated with anti-TNF agents revealed that 89% (233/262) of the patients with Behçet’s disease-associated uveitis experienced sustained remission following IFX therapy [35]. Two retrospective studies from Japan, each with a 10-year follow-up period, have confirmed the long-term efficacy of infliximab and its non-inferiority to traditional combination immunosuppressive therapy over an extended duration [36,37]. Research indicates that overall, the efficacy and safety profiles of IFX and ADA are comparable; however, IFX demonstrates a more rapid onset of action, rendering it particularly suitable for use during acute exacerbations [35,38,39,40]. Nonetheless, a small-scale prospective study focusing on pediatric refractory NIU revealed that after 40 months of follow-up, 60% (9/15) of patients receiving ADA maintained therapeutic remission, compared with only 18.8% (3/16) of those treated with IFX (*p* < 0.02) [41]. This finding underscores the superior efficacy of adalimumab in sustaining remission in chronic pediatric uveitis.

#### 3.2.3. Other Anti-TNF-α Antibodies

Currently, other anti-TNF-α antibodies, including golimumab and certolizumab pegol, are also considered to possess potential efficacy in patients with refractory NIU. However, the data supporting these treatments are derived from a small-sample, retrospective case series; thus, further investigations are warranted to evaluate their effectiveness comprehensively [16,42,43,44].

### 3.3. Non-Anti-TNF-α Monoclonal Antibodies

#### 3.3.1. Anti-Interleukin-6 Monoclonal Antibodies

Interleukin-6 (IL-6) is a pleiotropic inflammatory cytokine that modulates the function of various immune cells, including T cells, B cells, and macrophages [45,46]. In patients with refractory/chronic uveitis, IL-6 levels are typically elevated in both intraocular fluids and serum [46]. IL-6 exacerbates inflammatory responses by promoting Th17 cell differentiation and inhibiting regulatory T cell function while also stimulating B cells to produce autoantibodies [46]. In experimental autoimmune uveitis (EAU) mouse models with IL-6 knockout, uveitis development was significantly suppressed [45]. Furthermore, the systemic administration of anti-IL-6 receptor antibodies has been shown to ameliorate EAU by inhibiting both systemic and local Th17 responses [45]. These findings highlight the potential of IL-6 as a therapeutic target for uveitis. Tocilizumab (TCZ) is a humanized monoclonal antibody targeting the interleukin-6 receptor (IL-6R). Its primary mechanism of action involves binding with high specificity to both soluble and membrane-bound IL-6R, thereby inhibiting IL-6-mediated signaling and exerting potent anti-inflammatory effects [47]. The FDA has approved TCZ for the treatment of several conditions, including rheumatoid arthritis (RA), giant cell arteritis, and systemic juvenile idiopathic arthritis, underscoring its therapeutic relevance in managing inflammatory diseases [47,48]. In addition to its anti-inflammatory effects, research has demonstrated that inhibition of IL-6 significantly improves uveitic macular edema, which has raised considerable interest among ophthalmic specialists regarding the therapeutic potential of TCZ in the treatment of uveitis [7,16,49]. We conducted a small prospective study to evaluate the efficacy of TCZ in patients with NIU, excluding those with anterior uveitis. In this study, a total of 18 participants received intravenous infusions of TCZ at a dosage of 4 mg/kg, whereas 19 other participants were administered a higher dose of 8 mg/kg. At the six-month follow-up, both groups exhibited statistically significant improvements in best-corrected visual acuity (BCVA), with approximately 30% of patients demonstrating an enhancement of two or more lines on the visual acuity chart. Additionally, a notable reduction in the central macular thickness (CMT) was observed. Furthermore, among twenty-three patients who had baseline vitreous opacities that could potentially be reduced by two grades, ten patients (43.4%) experienced a decrease in opacity levels by two grades at the six-month mark. The authors also noted that systemic and ocular adverse events related to the primary endpoint were relatively limited [49]. Two small-scale retrospective studies demonstrated the efficacy of TCZ in treating refractory uveitis associated with juvenile idiopathic arthritis (JIA) [50] and Behçet’s disease [51]. In both studies, patients exhibited statistically significant improvements in visual acuity and reductions in CMT.

#### 3.3.2. Anti-CD20 Monoclonal Antibodies

CD20 is a transmembrane protein predominantly expressed on B cells that plays a crucial role in their development, activation, and differentiation [52]. Although T cell-mediated immune responses have traditionally been regarded as the predominant factor in the pathogenesis of uveitis, emerging evidence indicates that autoantibodies produced by B cells targeting ocular tissues have been identified in various forms of uveitis, suggesting a significant role for B cells in the development of NIU [52,53].

Rituximab (RTX) is a fully humanized chimeric anti-CD20 monoclonal antibody that depletes B cells. It has been approved by the FDA for the treatment of several conditions including non-Hodgkin’s lymphoma (NHL), chronic lymphocytic leukemia (CLL), RA, microscopic polyangiitis (MPA), and granulomatosis with polyangiitis (GPA) [54]. The efficacy of RTX in the treatment of corticosteroid-resistant and traditional-immunotherapy-refractory NIU has been substantiated in numerous reports. A meta-analysis consolidated individual data from 108 patients with refractory NIU who underwent treatment with RTX prior to 2021. The authors noted that rituximab therapy elicited a favorable therapeutic response in 83.5% (81/97) of NIU patients, with 76.3% (74/97) of cases reporting no adverse effects [54]. Two retrospective studies included a total of nine [55] and five [56] patients with uveitis due to Vogt–Koyanagi–Harada (VKH) disease, respectively; both groups were unresponsive to standard combination immunosuppressive therapy. In both studies, patients achieved remission and demonstrated significant visual improvement, and no complications associated with rituximab were observed.

#### 3.3.3. Other Non-Anti-TNF-α Monoclonal Antibodies

Other mAbs also exhibit therapeutic potential in uveitis. Emerging research on the IL-23/IL-17 axis in experimental models of NIU and autoimmune systemic or ocular disorders has highlighted the potential of targeting these cytokines as a therapeutic strategy for NIU [57,58]. Ustekinumab, which targets the shared p40 subunit of IL-23 and IL-12, has been reported in case studies to be effective in treating refractory uveitis associated with Behçet’s disease and psoriatic arthritis [59,60]. Currently, relevant clinical trials are underway to further investigate the efficacy of ustekinumab [7]. Secukinumab (AIN457) specifically targets IL-17A [61]. In an open-label study involving 16 patients diagnosed with active uveitis at baseline, it was observed that following two intravenous infusions of secukinumab (10 mg/kg), 13 patients (81%) exhibited, at minimum, a one-grade reduction in ocular inflammation [61].

## 4. Monoclonal Antibody Therapy for Age-Related Macular Degeneration

### 4.1. Age-Related Macular Degeneration

Age-related macular degeneration (AMD) is a widespread ophthalmic disorder that primarily affects the visual acuity of elderly people, with an estimated 14 million individuals affected globally [62]. It is one of the leading causes of central vision loss in individuals aged ≥ 60 years [63]. AMD primarily affects the macular region of the retina, which is responsible for sharp central vision and detailed perception. The condition is classified into two main types: dry (non-exudative) AMD and wet (exudative) AMD. Dry AMD typically progresses slowly, leading to mild to moderate vision impairment. Currently, no specific interventions are available for the treatment of dry AMD. Wet AMD, also referred to as exudative or neovascular AMD (NVAMD), affects over 15 million individuals worldwide, accounting for approximately 5% of the population aged 70 and older [62,64]. This condition is characterized by the emergence of choroidal neovascularization (CNV), a process in which newly formed blood vessels may exhibit leakage of fluid or experience hemorrhage, ultimately leading to retinal damage [62].

The underlying etiology primarily involves an imbalance between vascular endothelial growth factor (VEGF) and anti-angiogenic factors. In the context of NVAMD, retinal pigment epithelial cells, along with other retinal cell types, produce an excess of VEGF, which subsequently stimulates the formation of neovascular structures [63,65]. Based on the role of VEGF in NVAMD, anti-VEGF therapies have emerged as the primary treatment modality for this condition. These pharmacological agents function by inhibiting the activity of VEGF, thereby reducing the formation and leakage of neovascularization, protecting the retina, and mitigating the progression of vision loss [63,65,66].

### 4.2. Treatment of Age-Related Macular Degeneration with Anti-VEGF Monoclonal Antibodies

The VEGF family comprises five distinct proteins: VEGF-A, VEGF-B, VEGF-C, VEGF-D, and placental growth factor (PlGF). Each variant of VEGF is characterized by its specific receptor-binding affinity [67]. Among these, VEGF-A has been extensively studied and is considered the most significant because of its role as the principal angiogenic factor, often simply referred to as VEGF [67,68]. This factor exerts its biological function in the form of a homodimer composed of two identical subunits [67,68]. It stimulates endothelial cells through a series of intricate signal transduction pathways that are initiated when VEGF interacts with its primary receptors on the cell surface, namely, VEGF Receptor 1 (VEGFR-1) and VEGF Receptor 2 (VEGFR-2) [68]. Anti-VEGF therapies encompass several distinct categories. First, agents such as bevacizumab (Avastin) and ranibizumab (Lucentis) specifically target VEGF ligands by binding directly to them, thereby inhibiting their interaction with receptors on the surface of endothelial cells. Second, small-molecule tyrosine kinase inhibitors, such as sunitinib (Sutent), focus on the tyrosine kinase activity of VEGF receptors (VEGFR-1 and VEGFR-2), effectively obstructing downstream signaling pathways that are crucial for angiogenesis. Third, fusion proteins such as aflibercept (Eylea) function as decoy receptors by mimicking VEGF receptors. These proteins exhibit high binding affinity for VEGF ligands, sequestering them and preventing the activation of actual VEGF receptors on endothelial cells [67,69,70]. Table 2 compares the characteristics and efficacy of VEGF inhibitors in the treatment of NVAMD.

#### 4.2.1. Aflibercept

Aflibercept (Eylea) was engineered by combining the extracellular domains of human VEGF receptors 1 and 2 with the Fc domain of human IgG1. This unique structural configuration enables Eylea to bind with high affinity to VEGF-A, VEGF-B, and PlGF [66,69,102]. Eylea effectively sequesters these growth factors by functioning as decoy receptors, thereby preventing their interaction with native receptors on endothelial cells. This mechanism subsequently inhibits angiogenesis and modulates vascular permeability [103]. The FDA granted approval for VEGF Trap-Eye (Eylea), an intravitreal formulation of Eylea, in 2011 for the treatment of DME secondary to NVAMD [102,103]. Although it is not strictly a monoclonal antibody, Eylea’s inclusion in this review is warranted because of its current extensive application in ophthalmology and its similarity in therapeutic approach to antibody-based treatments.

In a Phase I trial examining the efficacy of intravitreal injection of Eylea for the treatment of CNV associated with neovascular NVAMD, the maximum tolerated dose was established at 1.0 mg/kg [104]. Administration of this dosage, whether as single or multiple injections, resulted in an approximate reduction of 60% in excess retinal thickness. However, among the five patients treated with a dosage of 3.0 mg/kg, two experienced dose-limiting toxicities that necessitated their withdrawal from the study [104]. This outcome prompted researchers to explore intravitreal administration of Eylea in subsequent trials. The subsequent Phase I study on intravitreal delivery of VEGF Trap-Eye in patients with NVAMD aimed to evaluate the safety, tolerability, maximum tolerated dose, and bioactivity of this treatment [105]. A total of 21 eligible NVAMD patients received a single intravitreal injection of VEGF Trap-Eye at doses of 0.05 mg, 0.15 mg, 0.5 mg, 1 mg, 2 mg, or 4 mg. At the six-week mark, the mean reduction in central foveal thickness across all patients was 104.5 μm, coupled with an average improvement of 4.43 letters in visual acuity. Notably, the highest dosage groups (2 mg and 4 mg) exhibited significant enhancements in BCVA, correlating with reductions in foveal thickness [105]. Furthermore, no severe adverse events or identifiable intraocular inflammation were reported by the conclusion of the twelve-week follow-up period, underscoring the favorable tolerability profile associated with intravitreal administration of Eylea [105].

#### 4.2.2. Bevacizumab

Bevacizumab (Avastin) is a fully humanized monoclonal antibody that encompasses two binding sites capable of interacting with VEGF-A [68]. Avastin was initially approved for the treatment of colorectal cancer and has been utilized, off label, for the management of NVAMD since 2005 [69,106]. A clinical study conducted in 2005 employed an open-label, single-center, non-comparative design to evaluate the safety of Avastin in patients with choroidal neovascularization (CNV) associated with AMD over a period of 24 weeks [107]. The study assessed changes in BCVA scores and optical coherence tomography (OCT) measurements relative to baseline. Patients received intravenous infusions of 5 mg/kg every two weeks, with one or two doses administered per session. The results indicated that no severe ocular or systemic adverse events were observed during the 24-week follow-up period. Furthermore, by week 24, the mean BCVA among the 18 patients improved by an average of 14 letters compared to baseline, while central retinal thickness demonstrated a mean reduction of 112 µm. This study affirmed the tolerability and effectiveness of systemic Avastin therapy for neovascular AMD, highlighting its enduring efficacy [107]. A retrospective case series was conducted to investigate the safety and efficacy of intravitreal injections of Avastin [108]. The study involved 79 patients with NVAMD, encompassing a total of 81 eyes receiving monthly Avastin intravitreal injections (1.25 mg) until resolution of macular edema, subretinal fluid (SRF), and/or pigment epithelial detachment (PED). At the 8-week follow-up, an evaluation of 51 eyes revealed complete resolution of retinal thickening, SRF, and PED in 25 eyes. Additionally, the mean central retinal thickness in the central 1 mm region decreased by 89 μm. Visual acuity demonstrated significant improvement, with median vision enhancing from 20/200 to 20/80 at 4 weeks and further progressing to similar levels by the end of the study period. No significant ocular or systemic adverse effects were observed at week 12 [108]. To date, the efficacy and tolerability of intravitreal Avastin have been extensively documented. Current consensus suggests that, for most patients, intravitreal injections of Avastin and ranibizumab exhibit comparable efficacy [68,109]. However, Avastin offers a significant cost advantage [68].

#### 4.2.3. Ranibizumab

Ranibizumab (Lucentis) is a recombinant humanized monoclonal antibody fragment specifically designed to bind and inhibit all identified isoforms of VEGF [68]. Lucentis is specifically engineered for intravitreal injection and has been approved by the FDA for the treatment of various ocular disorders, including NVAMD, diabetic macular edema (DME), retinal vein occlusion (RVO), and myopic CNV (mCNV). Compared to Avastin, the reduced volume facilitates superior penetration into the retinal layers. A design tailored explicitly for intraocular application further diminishes the risk of potential complications [68,69,110]. In a Phase III study titled MARINA, researchers assessed the efficacy of Lucentis for treating minimally classic or occult CNV devoid of classic features associated with AMD [111]. In this two-year, multi-center, double-blind, placebo-controlled study, a total of 716 patients with NVAMD were recruited and randomized in a ratio of 1:1:1. The participants received intravitreal injections of either 0.3 mg or 0.5 mg of Lucentis or placebo. In terms of visual acuity improvement, compared with the placebo group, the average gains associated with Lucentis were approximately 17 letters at the 12-month mark and ranged from 20 to 21 letters at the 24-month follow-up. Regarding safety profiles, both dosage groups of Lucentis exhibited a low incidence of severe ocular and systemic adverse events [111].

#### 4.2.4. Brolucizumab

Brolucizumab (Beovu) is a humanized monoclonal single-chain antibody fragment that specifically binds all VEGF-A isoforms [112]. It has been approved by the FDA and European Medicines Agency (EMA) for the treatment of NVAMD [113]. Beovu is a single-chain antibody fragment with a molecular weight of approximately 26 kDa, which makes it the smallest anti-VEGF agent currently available. This small size contributes to its high solubility and allows a more concentrated formulation, enabling the delivery of a higher dose in a smaller injection volume compared with other anti-VEGF therapies [82]. This allows for the delivery of a higher concentration of the drug; specifically, 6 mg of Beovu can be administered in a 50 µL injection, which is significantly more concentrated than Eylea [112]. The HAWK and HARRIER trials were pivotal Phase III, multicenter, double-blind studies conducted over 96 weeks to compare the efficacy and safety of Beovu with Eylea in patients with NVAMD [12]. In these trials, IVT injections of Beovu (3 mg or 6 mg in HAWK; 6 mg in HARRIER) were initially administered as loading doses every 4 weeks for three doses, followed by maintenance doses every 12 weeks, with the option to adjust to every 8 weeks if disease activity was detected. Eylea was administered according to its label at the time, with three initial monthly doses followed by injections every 8 weeks [12]. Both trials demonstrated that Beovu, administered every 12 or 8 weeks, was non-inferior to Eylea administered every 8 weeks in terms of changes in BCVA from baseline [12]. Regarding adverse events, the incidence of intraocular inflammation associated with Beovu, as reported in the HAWK and HARRIER trials, was 4.6% compared with 1.5% for Eylea. The incidence of severe vision loss was comparable between the two groups [12]. Additionally, following the approval of Beovu and its introduction to the market, post-market surveillance identified adverse events, such as retinal vasculitis and retinal vascular occlusion [114]. These findings prompted the FDA to update the product label in 2020 to include warnings and precautions regarding these potential risks [115].

#### 4.2.5. Faricimab

Faricimab (Vabysmo) is a bispecific IgG1 antibody designed to simultaneously bind and inhibit both VEGF-A and angiopoietin-2 (Ang-2). It has an overall molecular size of approximately 150 kDa [116]. Structurally, Vabysmo consists of two antigen-binding fragments (Fab), each targeting Angiopoietin-2 (Ang-2) and VEGF-A and a modified fragment crystallizable (Fc) region. Ang-2 is a key regulator of vascular remodeling and inflammation. It acts as an antagonist of Ang-1 by binding to the Tie2 receptor on endothelial cells, disrupting the stabilizing effects of Ang-1. This disruption leads to increased vascular permeability and inflammation, contributing to pathological angiogenesis in diseases such as NVAMD and DME [117]. By inhibiting Ang-2, Vabysmo restores vascular stability and reduces pathological neovascularization [116]. The dual-targeting approach aims to enhance therapeutic efficacy by addressing two distinct pathways involved in pathological angiogenesis and vascular instability [116]. A Phase II clinical trial, comprising the 36-week AVENUE and 40-week STAIRWAY studies, assessed the efficacy of Vabysmo compared to Lucentis in patients with NVAMD [90,118]. The AVENUE trial included 263 treatment-naïve patients with CNV secondary to NVAMD [118]. Participants were randomly assigned to one of the following treatment groups: monthly Lucentis 0.5 mg, monthly Vabysmo 1.5 mg, monthly Vabysmo 6 mg, bi-monthly Vabysmo 6 mg, or a sequential treatment of Lucentis 0.5 mg every 4 weeks until week 8 followed by Vabysmo 6 mg every 4 weeks. After 36 weeks, patients receiving a 1.5 mg monthly dose of Vabysmo showed a gain of +9.1 letters in BCVA. Those receiving monthly and bi-monthly doses of Vabysmo 6.0 mg improved by +6.0 letters, while patients on monthly Vabysmo 6.0 mg exhibited a gain of +5.9 letters. Finally, the group receiving Lucentis monthly experienced a gain of +7.2 letters. All treatment groups demonstrated anatomical improvements, with the most significant reduction in central retinal thickness (CRT) reduction observed in the sequential treatment group [118]. In the STAIRWAY trial, at week 40, vision gains from baseline were +11.4 letters (80% CI, 7.8–15.0) for the Lucentis every 4 weeks group, +9.3 letters (80% CI, 6.4–12.3) for the Vabysmo every 12 weeks group, and +12.5 letters (80% CI, 9.9–15.1) for the Vabysmo every 16 weeks group [90]. Anatomically, patients receiving Vabysmo every 12 and 16 weeks exhibited significant reductions in CRT, with changes in fluorescein angiography (FA)-measured total lesion area comparable to those observed in patients receiving monthly Lucentis [90]. Two Phase III non-inferiority trials, TENAYA and LUCERNE, evaluated the 2-year efficacy, durability, and safety of 6 mg Vabysmo compared to 2 mg Eylea in the treatment of NVAMD [91]. The findings indicated that Vabysmo 6 mg maintained vision gain in the second year under a treat-and-extend regimen based on nAMD disease activity, with most patients achieving extended dosing intervals. Its safety and efficacy were non-inferior to those of 2 mg Eylea [91].

## 5. Treatment of Other Retinal Diseases with Anti-VEGF Monoclonal Antibodies

### 5.1. Diabetic Retinopathy

Diabetic retinopathy (DR) is a prevalent microvascular complication of diabetes, affecting approximately 30–40% of individuals with this condition and is a leading cause of blindness among working-age adults [119]. DME is one of the most prevalent complications associated with DR and represents the primary cause of vision impairment among affected individuals [119,120]. Characterized by progressive damage to the retinal microvasculature resulting from chronic hyperglycemia, DR encompasses a spectrum of pathological changes, including microaneurysms, hemorrhages, exudates, capillary occlusion, and neovascularization [119,121]. Traditionally, the management of diabetic retinopathy (DR) has predominantly relied on laser photocoagulation and lifestyle modifications aimed at controlling blood glucose and blood pressure levels [121,122]. In contemporary practice, anti-VEGF agents have emerged as the standard treatment for DME and extensive randomized clinical trials have demonstrated their efficacy and safety [119,123,124].

Lucentis and Eylea were approved by the FDA in 2017 and 2019, respectively, for intravitreal injection in the treatment of DR [125,126]. Additionally, Avastin has been utilized off label for intravitreal injection in the management of DME [125]. A large multicenter, randomized clinical trial conducted in the United States compared the relative efficacy and safety of intravitreal injections of Eylea, Avastin, and Lucentis for the treatment of DME [126]. A total of 660 adults with center-involvement diabetic macular edema were randomly allocated in a ratio of approximately 1:1:1 to receive intravitreal Eylea at a dosage of 2.0 mg, Avastin at 1.25 mg, or Lucentis at 0.3 mg, administered once every four weeks. This study monitored changes in visual acuity over a one-year period. The results indicated that when the initial visual acuity was relatively preserved, there were no significant differences in the average outcomes among the treatment groups. Conversely, in instances where the initial visual acuity was compromised, Eylea demonstrated superior efficacy in enhancing vision. Furthermore, there were no notable differences in the safety profiles of the various treatment groups [126].

In 2022, the FDA approved Beovu for the treatment of DME [127]. The Phase III KITE and KESTREL trials investigated the one-year non-inferiority of 6 mg Beovu to 2 mg Eylea in the treatment of DME [128]. The results indicated that at week 52, 6 mg Beovu was non-inferior to Eylea in terms of the mean change in BCVA from baseline (KITE: +10.6 letters; KESTREL: +9.2 letters vs. +10.5 letters vs. +9.4 letters; *p* < 0.001). Additionally, studies showed that a greater number of subjects achieved a central subfield thickness (CST) of less than 280 µm following an initial series of five injections, with subsequent dosing intervals of 8 to 12 weeks [128].

The 36-week BOULEVARD Phase II trial enrolled 229 treatment-naive patients with DME [129]. Participants were randomly assigned to one of three groups to receive monthly treatments for a total of 20 weeks: 6.0 mg Vabysmo, 1.5 mg Vabysmo, or 0.3 mg Lucentis. The primary endpoint was assessed at 24 weeks with a follow-up period of 36 weeks. The 6.0 mg Vabysmo group demonstrated significant improvements in visual acuity, reduction in central subfield thickness, and improvement in the Diabetic Retinopathy Severity Scale (DRSS) compared to the other treatment groups, with no new or unexpected safety signals observed [129]. The identically designed Phase III clinical trials, YOSEMITE and RHINE, compared the one-year non-inferiority of 6.0 mg Vabysmo to 2.0 mg Eylea for the treatment of center-involved DME [130]. The trials demonstrated that 6.0 mg Vabysmo administered every 8 weeks achieved non-inferiority in terms of the primary endpoint—mean change in BCVA at one year—and safety compared to 2.0 mg Eylea administered every 8 weeks [130]. Based on the results from the Phase III trials, the FDA approved Vabysmo for DME treatment [131].

### 5.2. Retinal Vein Occlusion

Retinal vein occlusion (RVO) is a prevalent ocular vascular disorder that can cause significant visual impairment. Depending on the location of the occlusion, RVO is categorized into two distinct types: central retinal vein occlusion (CRVO) and branch retinal Vein occlusion (BRVO) [132,133]. The principal pathological alterations associated with RVO include retinal edema, hemorrhage, hypoxia, and neovascularization. The underlying mechanisms contributing to this condition are closely linked to VEGF overexpression [133]. Anti-VEGF therapy has emerged as a first-line treatment option for RVO by mitigating vascular permeability and inhibiting neovascularization, thereby ameliorating retinal edema and hypoxic conditions [132,133]. Lucentis was approved in June 2010 for the treatment of macular edema following both CRVO and BRVO based on the results of the BRAVO (BRVO) and CRUISE (CRVO) Phase III clinical trials [134,135]. Eylea received FDA approval for the treatment of macular edema secondary to CRVO in September 2012 and for macular edema secondary to BRVO in October 2014 based on the outcomes of the COPERNICUS (CRVO), GALILEO (CRVO), and VIBRANT (BRVO) Phase III clinical trials [136,137,138].

On the other hand, Avastin has not been approved by the FDA for the treatment of RVO to date. Although some clinical studies have demonstrated the efficacy of Avastin in the management of RVO, its use in this context remains off-label [132,139].

### 5.3. Retinopathy of Prematurity

Retinopathy of prematurity (ROP) is a condition that affects the retina of premature infants and is a leading cause of blindness in this vulnerable population worldwide [140,141]. The primary pathology of ROP is characterized by abnormal retinal vascular development, which is primarily caused by premature birth [140]. Traditionally, the standard treatment for ROP primarily involves destructive interventions targeting the avascular peripheral retina, predominantly laser therapy [140,141]. The recent consensus guidelines for ROP screening, jointly issued by the American Academy of Pediatrics (AAP) and the American Academy of Ophthalmology have incorporated the use of intravitreal anti-VEGF agents into the therapeutic recommendations for ROP [141,142]. In 2023, intravitreal injection of Eylea was approved in the United States for the treatment of severe ROP [141]. The FIREFLEYE Phase III randomized clinical trial, involving 27 countries, compared the non-inferiority of intravitreal injection of 0.4 mg Eylea to laser therapy in infants diagnosed with severe ROP [143]. A total of 113 infants were enrolled in the study, of whom 75 received Eylea and 38 underwent laser treatment. At the 24-week mark, the treatment success rate for Eylea was 85.5% (90% CrI: 78.0% to 91.3%), while the success rate for laser therapy was 82.1% (90% CrI: 70.5% to 90.8%). The conclusion drawn from this trial indicates that intravitreal Eylea did not meet the non-inferiority criteria when compared to laser treatment [143]. Subsequently, the FIREFLEYE Next study, which followed the FIREFLEYE trial, assessed the ophthalmic and safety outcomes of patients over a two-year period [5]. The findings indicated that at the age of two, 61 out of 63 (96.8%) patients in the Eylea group did not exhibit any signs of ROP, while 30 out of 32 (93.8%) patients in the laser group showed no evidence of ROP. Notably, there were no instances of late-stage retinal detachment observed in the Eylea group, and visual function was found to be age-appropriate. Furthermore, the incidence of myopia following intravitreal Eylea treatment was rare and showed no significant difference when compared to outcomes after laser therapy. Additionally, there are no drug-related safety issues concerning growth and neurodevelopmental results following intravitreal Eylea administration [5].

In 2019, the European Medicines Agency granted approval for the use of Lucentis in the treatment of severe retinopathy of prematurity (ROP) based on the findings of the Phase III trial entitled “The Lucentis compared with laser therapy for the treatment of Infants Born prematurely with ROP” (RAINBOW) [141,144]. The researchers concluded that, in the context of ROP treatment, Lucentis at a dosage of 0.2 mg demonstrated a superior success rate compared to laser therapy (80% versus 66%), resulted in fewer adverse ocular outcomes, and exhibited an acceptable safety profile over a 24-week period [144].

Numerous studies have underscored the beneficial effects of intravitreal Avastin in the treatment of ROP, and its therapeutic efficacy has been endorsed by the American AAP [141,142,145,146,147]. However, it is important to note that intravitreal injection of Avastin remains an off-label use [141].

It is noteworthy that long-term data on the potential systemic and neurodevelopmental impacts of anti-VEGF treatment for ROP are limited. Significantly, Avastin has been detected in serum within one day of intravitreal injection, with serum VEGF levels suppressed for at least 8 to 12 weeks [148,149]. The systemic absorption of anti-VEGF agents and their prolonged effect on serum VEGF levels raise concerns about potential off-target effects on organ development, particularly in the context of rapid growth and maturation occurring in premature infants [149]. Therefore, the temporary suppression of systemic VEGF levels could theoretically impact these processes, although the clinical significance of such effects remains unclear [150]. Given these considerations, the decision to use anti-VEGF therapy for ROP should be made on a case-by-case basis, carefully weighing the potential benefits against unknown long-term risks. Furthermore, large-scale, long-term follow-up studies are urgently needed to comprehensively assess the systemic effects and neurodevelopmental outcomes of anti-VEGF therapy in this vulnerable population.

## 6. Adverse Events of Intravitreal Anti-VEGF Injections

Certain complications following intravitreal injections of anti-VEGF agents are similar across pharmaceutical formulations. These adverse events may manifest after any intravitreal anti-VEGF injection and appear to be independent of the underlying pathological condition [151]. Common ocular adverse events associated with anti-VEGF antibody therapies included ocular pain, conjunctival hyperemia, elevated intraocular pressure, vitritis or vitreous detachment, visual disturbances, ocular myopathy, conjunctival hemorrhage, ocular irritation, foreign body sensation, increased lacrimation, blepharitis, dry eye, and ocular pruritus (Table 1). Less frequently, severe adverse events have been observed, including endophthalmitis, blindness, retinal detachment, retinal tears, and iatrogenic traumatic cataracts (Table 1) [151]. Infectious endophthalmitis remains one of the most devastating complications of intravitreal anti-VEGF injections. In multicenter clinical trials of anti-VEGF therapy, the reported incidence of endophthalmitis per patient ranged from 0.019% to 1.6% [151]. However, with the standardization of aseptic techniques following widespread clinical adoption of anti-VEGF intravitreal injections, the reported rates of infectious endophthalmitis have decreased compared to earlier cohorts [151,152]. The primary adverse event associated with Lucentis is ocular inflammation. In two Phase III clinical trials evaluating intravitreal Lucentis for AMD treatment, the incidence of significant ocular inflammation was reported to be 2.1% and 2.9%, respectively [111,153]. In contrast, the reported incidence of ocular inflammation following intravitreal Avastin injections is notably lower, ranging from 0.09% to 0.4% [153,154,155]. Eylea demonstrates an even lower rate of ocular inflammation, with an approximate incidence of 0.05% (14 logged out of 30,000 injections) per intravitreal administration [151].

A pivotal Phase III study comparing the safety profiles of Eylea and Lucentis in NVAMD enrolled 2419 patients with active treatment-naïve exudative AMD. Participants were randomized 1:1:1:1 to receive either Eylea 2 mg every 8 weeks (after a 3-month loading phase), Eylea (0.5 mg) every 4 weeks, Eylea 2 mg every 4 weeks, or Lucentis 0.5 mg every 4 weeks. The study found no significant differences in the incidence of severe ocular adverse events or systemic side effects between the treatment groups [138].

As for systemic adverse events, although intravitreal administration of low doses may reduce the risk of systemic adverse events, these drugs are ultimately cleared through systemic circulation [156,157]. Avastin has been detected in the untreated contralateral eye of rabbit models of NVAMD [156]. This raises concerns that serious systemic adverse events observed with intravenous Avastin in adjuvant chemotherapy, including proteinuria, hypertension, hemorrhage, and thromboembolic events, might occur in patients receiving intravitreal Avastin injections [153].

## 7. Treatment of Age-Related Macular Degeneration with Complement System-Targeting Antibodies

The complement system is a vital component of the innate immune response and circulates in an inactive zymogen form within the bloodstream. It can be activated through three distinct pathways: the classical, alternative, and lectin pathways [158]. Research has revealed that the activity of the alternative complement pathway in the eyes of patients with AMD is significantly elevated compared to that of healthy individuals. The excessive activation of this alternative pathway precipitates a sustained inflammatory response, resulting in damage to the RPE and Bruch’s membrane, thereby creating a conducive environment for CNV formation [158,159].

Moreover, this inflammatory milieu stimulates cells such as RPE to secrete VEGF, whereas complement activation products, including C3a and C5a, recruit macrophages and prompt them to release VEGF [62,159]. Conversely, VEGF can modulate complement activation by upregulating the expression of complement regulatory factors. This interplay engenders a vicious cycle that collectively advances the onset and progression of AMD [158,159,160].

### 7.1. Lampalizumab

Lampalizumab (FcFD4514S, mAb 166-32) is a humanized monoclonal antibody that works by inhibiting activation of the alternative complement pathway [62,160]. This is achieved by suppressing complement factor D, a key protease in the alternative pathway that helps cleave and activate complement factor B. This activation initiates a cascade reaction crucial for pathway function [160]. A Phase Ia study published in 2014 established the safety, tolerability, maximum tolerated dose, and immunogenicity of intravitreal injections of lampalizumab in 18 patients with geographic atrophy (GA) [161]. No dose-related toxicity or ocular or systemic adverse events were observed in the study cohort, with the maximum tolerated dose being the highest tested dose of 10 mg. The study confirmed that a single intravitreal injection of lampalizumab was safe and well tolerated [161]. The MAHALO Phase II randomized controlled trial enrolled 129 patients with bilateral GA but no choroidal neovascularization to evaluate the efficacy of intravitreal lampalizumab injections in patients with GA secondary to AMD [162]. Patients were randomized 1:2:1:2 into groups receiving monthly sham surgery, monthly 10 mg lampalizumab treatment, every-other-month sham surgery, or every-other-month 10 mg lampalizumab treatment. At the primary efficacy endpoint of 18 months, patients treated monthly with lampalizumab showed a 20% reduction in GA progression compared with sham surgery controls (80% confidence interval [CI], 4% to 37%). A more significant effect was observed in the subgroup of patients carrying the complement factor I risk allele, with a 44% reduction in GA progression compared with sham surgery controls. Additionally, lampalizumab demonstrated acceptable safety in this study [162].

Despite the promising outcomes observed in the Phase II trial, the subsequent Chroma and Spectri Phase III Randomized Clinical Trials enrolled 1881 eligible patients diagnosed with bilateral GA devoid of choroidal neovascularization [160]. This study aimed to assess the efficacy and safety of lampalizumab for GA secondary to AMD. Participants were randomized in a 2:1:2:1 ratio to receive either 10 mg of intravitreal lampalizumab administered every four weeks, sham surgery every four weeks, 10 mg of lampalizumab administered every six weeks, or sham surgery every six weeks for a duration of up to 96 weeks. Throughout the 48-week treatment period, lampalizumab failed to demonstrate any significant reduction in GA enlargement when compared to sham surgery [160]. These finding suggests that lampalizumab lacks effectiveness as a therapeutic intervention for GA associated with AMD.

### 7.2. Pegcetacoplan

Pegcetacoplan, a pegylated synthetic cyclic peptide, while not classified as a monoclonal antibody, demonstrates unique therapeutic value in ophthalmology through its mechanism as a complement C3 inhibitor [163]. In February 2023, pegcetacoplan received approval from the FDA as the first and only treatment for geographic atrophy (GA) secondary to AMD [164]. Although this review primarily focuses on mAbs, pegcetacoplan’s therapeutic principle shares similarities with certain antibody therapies, particularly in targeting specific molecules for ocular disease treatment. Therefore, we have included this information in our discussion.

Liao et al. conducted a randomized Phase II trial to evaluate the safety and efficacy of pegcetacoplan for the treatment of geographic atrophy (GA) [163]. In this study, 246 patients with GA were randomized in a 2:2:1:1 ratio to receive intravitreal injections of 15 mg pegcetacoplan monthly or every other month (EOM) or sham intravitreal injections monthly or EOM for 12 months. The results demonstrated that compared to sham treatment, pegcetacoplan reduced GA growth rates by 29% (95% confidence interval [CI], 9–49; *p* = 0.008) and 20% (95% CI, 0–40; *p* = 0.067) in patients receiving monthly or EOM pegcetacoplan, respectively [163]. In the subsequent Phase III OAKS and DERBY trials conducted by Heier et al., 1258 participants were randomized (2:2:1:1) to receive intravitreal injections of pegcetacoplan (15 mg) monthly or every other month or sham injections monthly or every other month for 24 months [164]. At 24 months, both OAKS and DERBY demonstrated a significant reduction in geographic atrophy progression with monthly and every-other-month pegcetacoplan compared to sham treatment. The incidence of new-onset exudative neovascular AMD at 24 months in the monthly pegcetacoplan, every-other-month pegcetacoplan, and sham groups was 11%, 8%, and 2% in OAKS and 13%, 6%, and 4% in DERBY, respectively. Regarding safety, based on pooled data from the OAKS and DERBY trials, the rate of infectious endophthalmitis per injection was 0.05% at 12 months and 0.03% at 24 months. Three serious adverse events consisting of ischemic optic neuropathy have been reported in patients receiving monthly pegcetacoplan for over 24 months [164].

### 7.3. Other Complement System-Targeting Antibodies

Eculizumab is a humanized monoclonal antibody targeting C5 that inhibits the formation of the membrane attack complex (MAC) by blocking the terminal pathway of complement activation [165]. It has received approval for the treatment of paroxysmal nocturnal hemoglobinuria (PNH) and atypical hemolytic uremic syndrome (aHUS) [166,167]. The inhibitory effects of eculizumab on choroidal neovascularization (CNV) have been confirmed in murine CNV models [168]. Nonetheless, there remains a paucity of large-scale clinical data to support these findings [169]. Additionally, the antibodies currently under development targeting the complement system include the monoclonal antibody ANX007, which is directed against C1q, and the monoclonal antibody NGM621, which targets C3 [170,171]. The former inhibits complement activation by obstructing the classical pathway, whereas the latter impedes C3 cleavage, thereby suppressing the convergence of all three activation pathways at the C3 level. Both antibodies are presently undergoing Phase II clinical trials for GA [172,173].

In summary, complement-targeted therapies for AMD have made significant strides in recent years, with several drugs at various stages of clinical development offering new hope for the treatment of GA in non-NVAMD patients.

## 8. Cost-Effectiveness Analysis of Intravitreal Antibody Therapy

Intravitreal mAb therapies have demonstrated remarkable efficacy in various ocular diseases; however, their high cost poses significant challenges in clinical applications.

In evaluating treatment cost-effectiveness, quality-adjusted life years (QALYs) are a crucial metric. A study comparing four treatment regimens for newly diagnosed NVAMD patients aged 80 years provided valuable insights [174]. The incremental cost-effectiveness ratio (ICER) for monthly Avastin compared to as-needed Avastin was $242,357/QALY. Notably, monthly Lucentis yielded only 0.02 additional QALYs compared with monthly Avastin, with an ICER exceeding $10 million/QALY [174]. These findings underscore Avastin’s superior cost-effectiveness in NVAMD treatment. However, the intravitreal use of Avastin remains controversial due to legal and safety concerns. Sensitivity analysis revealed that even under extreme assumptions favoring Lucentis, the as-needed regimen’s ICER was $97,340/QALY [2]. This emphasizes the importance of balancing costs and efficacy in treatment selection.

Monoclonal antibody therapy for uveitis has similar cost-effectiveness challenges. A study evaluating adalimumab for noninfectious intermediate, posterior, or pan-uveitis used a Markov model based on VISUAL I and II trial data [175]. For active uveitis, adalimumab’s ICER compared to limited current practice (LCP) was £92,600/QALY, while for inactive uveitis, it was £318,075/QALY. Sensitivity analyses showed potential ICER ranges of £15,579–£120,653/QALY for active uveitis and £35,642–£800,775/QALY for inactive uveitis [175]. The authors emphasized that these values significantly exceeded the cost-effectiveness thresholds typically recognized in the United Kingdom [175].

To address the challenge of reconciling efficacy with cost and accessibility, the industry is actively investigating a range of strategies aimed at enhancing the cost-effectiveness of mAb therapies. Novel delivery systems, such as the Port Delivery System with Lucentis (PDS), aim to reduce treatment-related visits by extending dosing intervals [70]. The introduction of biosimilars also offers hope for price reduction, with the Lucentis biosimilar Byooviz expected to be approximately 40% cheaper than the original [176]. However, these efforts face numerous obstacles. Developing new technologies requires substantial investment, potentially increasing short-term costs. Although biosimilars offer lower prices, their approval process is complex, and market acceptance remains uncertain. Moreover, differences in healthcare policies and insurance systems across countries affect the accessibility and cost-effectiveness of mAb therapies [176].

## 9. Conclusions

This review comprehensively elucidates the application of mAbs in the management of intraocular diseases including uveitis, AMD, diabetic retinopathy, and retinopathy of prematurity. Evidence indicates that anti-VEGF mAbs, including Avastin, Lucentis, and Eylea, demonstrate significant efficacy in treating neovascular AMD and diabetic macular edema. Concurrently, anti-TNF-α mAbs such as adalimumab and infliximab have also shown favorable outcomes in the treatment of NIU. Recently, complement system-targeted antibodies, such as pegcetacoplan, have made groundbreaking advances in the treatment of geographic atrophy, offering new therapeutic options for patients with non-neovascular AMD.

However, these therapeutic strategies are confronted with numerous challenges, including high treatment costs, potential issues related to drug resistance, and a lack of long-term safety and efficacy data. Future research should focus on the development of novel drug delivery systems to extend dosing intervals, explore combination therapies to enhance therapeutic effects, conduct large-scale longitudinal studies to assess long-term safety and effectiveness, investigate personalized treatment strategies to optimize therapeutic regimens, and develop biosimilars to reduce treatment costs. Furthermore, emerging complement system-targeted antibodies require additional investigation into their comparative efficacy with existing therapies and their optimal timing for application across different stages of disease.

In summary, while monoclonal antibody therapies exhibit immense potential in the field of ophthalmology, ongoing innovation coupled with rigorous clinical evaluation is essential for refining their application. This endeavor aims to provide safer, more effective, and more economically viable treatment options for patients in the future.

## Figures and Tables

**Table 1:** Summary of monoclonal antibodies for treating intraocular diseases.

Characteristics	Brand Name	Target	Molecular Weight	Structure	Administration	Ocular Indications	FDA Approval	Common Adverse Events	Serious Adverse Events	Link to the FDA Label
Ranibizumab	Lucentis	VEGF-A	48 kDa	Fab fragment	Intravitreal injection	- Wet AMD - DME - RVO - Myopic CNV	Yes	- Eye pain - Conjunctival hemorrhage - Vitreous floaters - Retinal detachment	- Endophthalmitis - Retinal detachment - Intraocular inflammation	https://www.accessdata.fda.gov/drugsatfda_docs/label/2006/125156lbl.pdf (accessed on 11 September 2024)
Bevacizumab	Avastin	VEGF-A	149 kDa	Full-length IgG1	Intravitreal injection	- Wet AMD - DME - RVO - Myopic CNV	Off-label use for eye diseases	- Eye pain - Conjunctival hemorrhage - Vitreous floaters - Retinal detachment	- Endophthalmitis - Retinal detachment - Intraocular inflammation	https://www.accessdata.fda.gov/drugsatfda_docs/label/2004/125085lbl.pdf (accessed on 11 September 2024)
Brolucizumab	Beovu	VEGF-A	26 kDa	scFv	Intravitreal injection	Wet AMD	Yes	- Eye pain - Conjunctival hemorrhage - Vitreous floaters - Retinal detachment	- Endophthalmitis - Retinal detachment - Intraocular inflammation	https://www.accessdata.fda.gov/drugsatfda_docs/label/2019/761125s000lbl.pdf (accessed on 11 September 2024)
Faricimab	Vabysmo	VEGF-A, Ang-2	149 kDa	Full-length IgG1	Intravitreal injection	- Wet AMD - DME	Yes	- Eye pain - Conjunctival hemorrhage - Vitreous floaters - Retinal detachment	- Endophthalmitis - Retinal detachment - Intraocular inflammation	https://www.accessdata.fda.gov/drugsatfda_docs/label/2022/761235s000lbl.pdf (accessed on 11 September 2024)
Infliximab	Remicade	TNF-α	149 kDa	Full-length IgG1	Intravenous infusion	- Uveitis - Scleritis - Optic neuritis	Off-label use for eye diseases	- Headache - Fatigue - Nausea - Rash	- Serious infections - Malignancies - Heart failure	https://www.accessdata.fda.gov/drugsatfda_docs/label/1998/inflcen082498lb.pdf (accessed on 11 September 2024)
Rituximab	Rituxan	CD20	144 kDa	Chimeric IgG1	Intravenous infusion	- Ocular mucous membrane pemphigoid - Ocular cicatricial pemphigoid	Off-label use for eye diseases	- Infusion reactions - Headache - Fatigue - Nausea	- Serious infections - Progressive multifocal leukoencephalopathy - Hepatitis B reactivation	https://www.accessdata.fda.gov/drugsatfda_docs/appletter/2017/761064Orig1s000ltr.pdf (accessed on 11 September 2024)
Adalimumab	Humira	TNF-α	148 kDa	Full-length IgG1	Subcutaneous injection	- Uveitis - Scleritis - Optic neuritis	Off-label use for eye diseases	- Injection site reactions - Upper respiratory infections - Headache	- Serious infections - Malignancies - Demyelinating disorders	https://www.accessdata.fda.gov/drugsatfda_docs/label/2011/125057s0276lbl.pdf (accessed on 11 September 2024)
Tocilizumab	Actemra	IL-6	148 kDa	Humanized IgG1	Intravenous infusion	- Uveitis - Scleritis	Off-label use for eye diseases	- Headache - Infections - Elevated liver enzymes	- Serious infections - Gastrointestinal perforations - Elevated liver enzymes	https://www.accessdata.fda.gov/drugsatfda_docs/label/2013/125472s000lbl.pdf (accessed on 11 September 2024)

**Table 2:** Comparison of characteristics and efficacy of VEGF inhibitors in NVAMD treatment.

Characteristics	Aflibercept	Ranibizumab	Bevacizumab	Brolucizumab	Faricimab
Binding affinity for VEGF-A_165_	0.5 pM [71]	46 pM [72]	58 pM [72]	28.4 pM [73]	3 nM [74]
Systemic half-life	5–6 days [75]	2 h [76]	20 days [75]	5.6 ± 1.5 h [77]	7.5 days [78]
Ocular half-life	9 days [79]	7.19 days [80]	4.9 days [81]	5.1 ± 2.78 days [82]	7.5 days [78]
Average 12-month BCVA improvement in NVAMD study trials (letters)	**VIEW1** IVT–0.5 mg–q4w: +6.9; IVT–2 mg–q4w: +10.9; IVT–2.0 mg–q8w: +7.9 [11]. **VIEW2**: IVT–0.5 mg–q4w: +9.7; IVT–2 mg–q4w: +7.6; IVT–2.0 mg–q8w: +8.9 [11] **ALTAIR** IVT–2 mg–3q4w/T&E–2W +9.0; IVT–AFL–2 mg–3q4w/T&E–4W +8.4 [83]. **HAWK (48 weeks)** IVT–2.0 mg–q8w: +6.8 [12]. **HARRIER (48 weeks)** IVT–2.0 mg–q8w: +7.6 [12]. **CANDELA (44 weeks)** IVT–8 mg–3q4w/fixed: +7.9; IVT–2 mg–3q4w/Fixed: +5.1 [84].	**CATT** IVT–0.5 mg–q4w: +8.5; IVT–0.5 mg–PRN: +6.8 [85]. **ANCHOR** IVT–0.3 mg–q4w: +8.5; IVT–0.5 mg–q4w: +11.3 [86]. **MARINA** IVT–0.3 mg–q4w: +6.5; IVT–0.5 mg–q4w: +7.2 [9]. **HARBOR** IVT–0.5 mg–q4w: +10.1; IVT–0.5 mg–PRN: +8.2; IVT–2.0 mg–q4w: +9.2; IVT–2.0 mg–PRN: +8.6 [9]. **TREX-AMD** IVT–0.5 mg–q4w: +9.2; IVT–0.5 mg–PRN: +10.5 [87]. **CANTREAT** IVT–0.5 mg–q4w: +6.0; IVT–0.5 mg–PRN: +8.4 [88].	**CATT** IVT–1.25 mg–q4w: +8.0; IVT–1.25 mg–PRN: +5.9 [85]. **ABC** IVT–1.25 mg–q4w: +7.0 [89]	**HAWK (48 weeks)** IVT–3 mg–q12w/q8w: +6.1; IVT–6 mg–q12w/q8w: +6.6 [12]. **HARRIER (48 weeks)** IVT–6 mg–q12w/q8w: +6.9 [12].	**STAIRWAY** IVT–6 mg–q12w: +10.1; IVT–6 mg–q16w: +11.4 [90].
Average 24-month BCVA improvement in study trials	**ALTAIR (96 weeks)** IVT–2 mg–3q4w/T&E–2W +7.6; IVT–AFL–2 mg–3q4w/T&E–4W +6.1 [83]. **TENAYA** IVT–6 mg–q8w: +3.3 [91]; **LUCERNE** IVT–6 mg–q8w: +5.2 [91].	**CATT** IVT–0.5 mg–q4w: +8.8; IVT–0.5 mg–PRN: +6.7 [92]; **ANCHOR** IVT–0.3 mg–q4w: +8.1; IVT–0.5 mg–q4w: +10.7 [93], **MARINA** IVT–0.3 mg–q4w: +5.4; IVT–0.5 mg–q4w: +6.6 [94], **HARBOR** IVT–0.5 mg–q4w: +9.1; IVT–0.5 mg–PRN: +7.9; IVT–2.0 mg–q4w: +8.0; IVT–2.0 mg–PRN: +7.6 [95], **TREND** IVT–0.5 mg–q4w: +7.9; IVT–0.5 mg–PRN: +6.6 [96]. **CANTREAT** IVT–0.5 mg–q4w: +6.0; IVT–0.5 mg–PRN: +6.8 [97].	**CATT** IVT–1.25 mg–q4w: +7.8; IVT–1.25 mg–PRN: +5.0 [92].	**MERLIN (recalcitrant nAMD)** IVT–6 mg–q4w: −0.8 [98].	**TENAYA** IVT–6 mg–q4w–q16w: +3.7 [91]; **LUCERNE** IVT–6 mg–q4w–q16w: +5.0 [91]
Single-dose cost	$1850 [99]	$1575 [100]	$50 [100]	$1418 [100]	$1350 [101]
